# Mothers balancing work and family: the associations with emotional well-being, sleep–wake problems and the role of basic needs

**DOI:** 10.1186/s40359-024-02241-3

**Published:** 2024-12-18

**Authors:** Rosalia Olivieri, Alessandro Lo Presti, Sebastiano Costa, Lucia Ariemma, Marco Fabbri

**Affiliations:** 1 Department of Psychology, Università Degli Studi Della Campania “Luigi Vanvitelli”, Viale Ellittico, 31, 81100 Caserta, Italy; 2 Department of Psychology “Renzo Canestrari”, Alma Mater Studiorum - Università di Bologna, Viale Berti Pichat 5, Bologna, 40127 Italy

**Keywords:** Basic Needs, Working Mothers, Sleep–wake problems, Work-Family Balance

## Abstract

**Background:**

The transition to motherhood involves significant changes, extending from pregnancy to the early years of a child's life. This period, characterized by multiple stressors and adjustments, can profoundly impact maternal well-being. For this reason, the aim of this study was to investigate the associations between work-family balance, need satisfaction, depression, anger, vitality and sleep–wake problems, using Self-determination Theory (SDT) as a theoretical framework.

**Methods:**

Participated in this study 218 working mothers in Italy with children aged between 1 and 36 months. Using a correlational design, SEM with latent variables was used to test an integrative model.

**Results:**

Results revealed significant positive associations between work-family balance and need satisfaction, and vitality, and negative direct associations of need satisfaction with sleep–wake problems, depression, and anger. Indirect associations were also visible between work-family balance and psychological outcomes through need satisfaction.

Conclusions.

Overall, findings underscore the importance of conditions that support the satisfaction autonomy, competence, and relatedness of working mothers during this critical life stage. These insights have implications for organizational policies and interventions aimed at supporting the mental health of working mothers.

In 2023, the female employment rate in Italy was just 56.5%, significantly lower than the European Union average of 70.2% [1]. This disparity is even more pronounced among mothers, with one in five women leaving the workforce after having children [2]. The primary reasons cited by women who leave their jobs post-maternity are related to the difficulty in balancing work and childcare [3]. Additionally, many working mothers report that their psychological well-being was higher before becoming mothers [4], and globally, working mothers express persistent concerns about their mental health [5].

Transition to motherhood is a particularly sensitive transformation process characterized by several bio-psycho-social changes [6] which typically begins with the awareness of becoming a parent and can persist until approximately the third year of age of child [7, 8]. In most cases, it represents a joyful event, but it is also accompanied by biological, psychological, social, economic, and behavioural changes that can be critical for adult health [9]. Mothers of newborns report insufficient sleep and may encounter significant sleep disruption [10] as childbirth causes pervasive sleep effects for women regardless of whether it is primiparous or multiparous [11]. Additionally, the sleep of both mothers and fathers does not return to pre-pregnancy levels for up to six years following the birth [11].

The birth of a child generally also leads to changes in emotional well-being. Mood disorders, in particular, have been one the most medical complication of pregnancy and post-partum period [12]. It is estimated that around 14% have post-partum depression with prevalence rates that vary worldwide [13]. Transition to motherhood can be accompanied by many tensions that may lead to feelings of anger especially in the life of the new mother [14]. Frequently, it is observed that parents experience fewer positive and more negative emotions than those who are not, suggesting that the implications of parenthood in everyday life are emotionally exhausting [15]. This is even more evident in mothers considering that in several cultural contexts, gender roles attribute greater responsibility for care compared to fathers, and this overload could hurt various aspects of subjective well-being [15, 16].

This becomes even more evident when work responsibilities are added to family obligations. Indeed, the resulting overload increases fatigue in parents [17]. Additionally, more women who continued to work after giving birth reported experiencing tensions between family and work demands [18]. Compared to the past, there have been substantial changes in workforce demographics with an increased number of women that are employed having highly increased work and family demands. Despite the shifting landscape of traditional role models, a gendered imbalance persists in household responsibilities, wherein employed women continue to shoulder many childcare and domestic tasks. This double role that an ever-increasing number of women has found themselves facing expresses the need to investigate how these two dimensions are reconciled and balanced. For this reason, the importance of work-family balance has emerged.

Work-family balance is a multidimensional construct that can be defined in terms of both the direction of influence between work and family roles (work-to-family versus family-to-work) and the type of effect (conflict versus facilitation) [19]. Research suggests two more precise meanings for work-family balance. The first meaning of work-family balance is a lack of conflict or interference between work and family roles, the second meaning refers to work-family facilitation (also referred to as work-family enhancement and positive work-family spillover) (e.g., [20, 21]). Although varying uses and definitions of the work-family balance term exist, the work-family balance could be defined as an overall appraisal regarding one's effectiveness and satisfaction with work and family life [22]. Work-family balance is increasingly important in today's society and has been associated with different indicators of well-being. Allen and Kiburz [23] have shown that sleep quality and vitality also positively relates to work-family balance. When individuals successfully manage the demands of both work and family life, they are likely to experience better sleep, reduced feelings of emotional exhaustion, fatigue, and lack of energy [24], as well as an enhanced sense of vitality. Achieving work-family balance can enhance overall energy levels and mental clarity, reduce psychological distress, emotional exhaustion, anger, and depression [24, 25] and increases life satisfaction [26], while incompatibility between work and family life has been associated with parental depression, poor physical health, poor health behaviours, and worse sleep quality [27]. In line with what emerged in previous literature, this study aims to confirm the association between work-family balance and anger, depression, vitality and sleep–wake problems also in working mothers, but also trying to delve deeper into the possible explanatory mechanisms (paths *c* of Fig. 1).

**Fig. 1:**
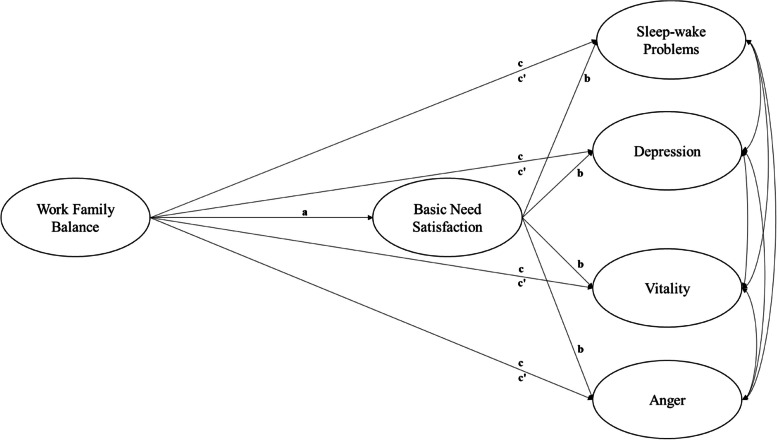
Hypothesized model. Note: Paths a, b, and c represent direct associations, while paths c’ are indirect associations

Considering the increase in the prevalence of working mothers and that the lack of balance between work and family can lead to ill-being it draws attention to the psychological mechanism that could explain the association between work and family and the psychological outcomes of working mothers. Although the literature has extensively demonstrated the positive role of work-family balance on individuals' mental health and quality of life [24], the underlying mechanisms remain underexplored. The application of the principles of Self-determination theory is gaining growing interest in the work-family literature [28–30]. From the perspective of self‐determination theory (SDT; 31), a healthy balance between work and family could represent a need-supportive condition because satisfy the need for autonomy, competence and relatedness. Autonomy refers to the experience of volition and willingness and a perceived sense of control over one’s actions, competence concerns the experience of effectiveness and mastery and describes a feeling of being capable of completing tasks and activity, relatedness indicates the experience of warmth, bonding, and care, and is satisfied by connecting to and feeling significant to others [31, 32]. Specifically, when there is a healthy balance between work and family, mothers may be able to voluntarily choose daily activities, manage to freely reconcile the tasks of both domains and feel a general sense of satisfaction with autonomy. Similarly, in a state of balance between work and family, working mothers will feel a greater sense of self-efficacy in their daily lives having the perception of being able to effectively face the challenges in both contexts in an effective manner (competence satisfaction). Finally, when work and family are in balance, working mothers are likely able to feel more involved in daily social dynamics and this could encourage greater availability of time to spend with family members and colleagues, giving the possibility of experiencing warmer relationships (relatedness satisfaction). Although this interpretation is plausible starting from the application of the basic principles of SDT, specific studies on work-family balance and needs are currently lacking, especially in a sample of working mothers with young children. For these reasons, this study aims to test the association between work-family balance and need satisfaction in working mothers after a period of maternity leave (paths *a* of Fig. 1).

However, a multitude of studies that have applied SDT have shown that satisfaction of these three needs promotes well‐being and reduces the risk of developing maladjustment [31, 32]. In the general population, the satisfaction of basic needs is positively associated with vitality [33] and negatively associated with anger [34], and depression [33]. Similarly, several studies have shown how low levels of need satisfaction are associated with sleep difficulties [35, 36]. However, the association between basic psychological needs and anger, depression, vitality and sleep–wake problems in working mothers is still not well understood and for this reason this study aims to explore these processes (paths *b* of Fig. 1).

Overall, several studies have highlighted also the mediating role of the satisfaction of needs in the association between contextual factors and indicators of mental health [31, 32] and the important role of psychological basic needs for emotional well-being of mothers (i.e., 37, 38) and workers (i.e., 29, 30). However, less attention has been paid to the associations between work-family balance, basic needs and indicators of emotional health and sleep–wake problems in working mothers in the first years after the pregnancy which is usually characterized by the returning to work after a variable period of break due to childbirth. This is particularly relevant in the Italian context given that recent statistics point out that in 2022 working mothers of children aged 0–3 years represent 73% of all voluntary resignations from work, compared to the 27% of fathers with children of the same age [39]. In light of all these considerations, this study therefore aims to verify the indirect association through need satisfaction of the association between the work-family balance and anger, depression, vitality and sleep–wake problems (paths *c’* of Fig. 1).

## The present study

This study offers a novel contribution to the field by applying SDT to the context of working mothers in the early years following pregnancy. While existing literature has extensively examined work-family balance and its impact on well-being, there remains a notable research gap regarding the psychological processes underlying this dynamic. The innovative aspect of this study is its focus on SDT and the crucial role of basic psychological needs in the relationship between work-family balance and outcomes such as depression, anger, vitality, and sleep–wake issues. By providing new insights into the mechanisms driving these processes, this research is vital for informing interventions and support systems for working mothers. It also has the potential to guide the development of policies and resources that enhance mothers' well-being and improve their overall quality of life.

Following previous consideration, the general aim of this study is to test a general model (Fig. 1) based on the principle of SDT to test the associations between work-family balance, need satisfaction, depression, anger, vitality and sleep–wake problems in working mothers with a child within three years of age. Specifically, the first aim was to test the direct association between work-family balance and need satisfaction (path *a* of Fig. 1). The second aim was to test the associations between need satisfaction and anger, depression, vitality and sleep–wake problems (paths *b* of Fig. 1). The third aim was to test the direct and indirect (through need satisfaction) associations between the work-family balance and anger, depression, vitality and sleep–wake problems (paths *c* and *c’* of Fig. 1).

## Method

### Study design

A crosse sectional study based on a correlational design was used. A convenience sample was enrolled voluntarily in the Italian territory, the majority residing in the Campania region. The participants did not receive any form of compensation for participation. The minimum sample size to detect a medium effect was of 161 participants and it was determined with the online a-priori sample size calculator for structural equation models [40] based on 6 latent variables and 21 indicators for a desired statistical power level of 0.08 and probability level at 0.05. Inclusion criteria were to be working mothers, age of children between (1) and (36) months. Exclusion criteria were to not be the biological mother, not having had a pregnancy in the last 3 years; not having returned to work after the birth of the child. The researchers recruited the working mothers through their social groups or a social network (Facebook or Instagram), through direct acquaintances, and contacted several local nurseries. In some cases, the participants distributed the survey among their friends, creating something similar to a snowball approach. This type of sampling helped reach a more diverse sample of participants. Therefore, given the nature of the convenience sample, it is not known how many participants were contacted but declined to participate. Inclusion and exclusion criteria were verified based on participants' self-completion of specific questions. Before completing the questionnaires, the participants read and signed the informed consent. Working mothers filled out an online questionnaire through a weblink provided by PsyToolkit [41, 42]. This link was sent via email, social networks or WhatsApp. In some cases, the participants were also provided with flyers on which the QR code of the questionnaire or the link was visible. To protect the privacy of the participants, the responses received were collected in an online dataset without the possibility of knowing their identity. The research was approved by the author’s university ethics committee and was conducted in accordance with the Declaration of Helsinki, the American Psychological Association (APA) criteria and the Ethical code of the Italian Psychological Association (AIP).

### Participants

This study included 218 Italian working mothers (Table 1), aged 21 to 50 years (M = 34.64, SD = 5.13), who had returned to work after pregnancy. They worked between 2 and 54 h per week (M = 27.92, SD = 11.62) and had at least one child aged between 1 and 36 months (M = 15.44, SD = 9.38). Regarding educational qualifications, 9 working mothers (4%) had a middle school certificate, 70 (32%) a high-school diploma, 111 (51%) a university degree, 26 (12%) a post graduate certification, and 2 (1%) reported “other”. Regarding marital status, 9 of the women (4%) were not in a stable relationship, 7 (3%) were in a stable relationship, 151 (69%) were married, 49 (23%) were cohabiting, and 2 (1%) were divorced. In the professional category, 169 mothers (78%) reported being “employees”, while only 43 (20%) indicated “private/Freelance”, and 6 (2%) reported “other”. 125 (57%) working mothers reported “working full-time”, while 57 (26%) reported working “part-time”, and finally, 36 (17%) mothers reported “other”. In regard to the professional field, 7 workers (3.2%) were in the primary sector, the secondary sector comprises 39 workers (18%), 117 workers reported to be in the tertiary sector (54%), while 55 workers (25%) fall under 'other' sectors category. Reporting information about the experience of the pregnancy, 188 mothers (86%) indicated having a “full term pregnancy”, followed 17 mothers (8%) who had “preterm pregnancy” and 13 (6%) that reported “protracted pregnancy”. Furthermore, 113 mothers (52%) reported breastfeeding, followed by 72 mothers (33%) that reported breast and bottle feeding, 31 (14%) mothers reported only bottle feeding while 2 (1%) reported “other”. Finally, the data revealed 121 working mothers (56%) were primiparous, followed 73 (33%) working mothers who had 2 children, 13 (6%) working mothers who had 3 children, only one mother (1%) with 4 children, and 10 participants (4%) did not answer to this question.

**Table 1:** Demographic characteristics of the sample

		N (%)	M (SD)
Age			34.64 (5.13)
Age of children			15.44 (9.38)
Weekly hours of work			27.92 (11.62)
Education			
	Middle school diploma	9 (4%)	
	High school diploma	70 (32%)	
	University Degree	111 (51%)	
	Post graduate	26 (12%)	
	Other	2 (1%)	
Marital Status			
	Not in a stable relationship	9 (4%)	
	In a stable relationship	7 (3%)	
	Married	151 (69%)	
	Cohabitants	49 (23%)	
	Divorced	2 (1%)	
Professional category			
	Employees	169 (78%)	
	Private/Freelance	43 (20%)	
	Other	6 (2%)	
Working time			
	Working full-time	125 (57%)	
	Working part-time	57 (26%)	
	Other	36 (17%)	
Professional field			
	Primary sector	7 (3%)	
	Secondary sector	39 (18%)	
	Tertiary sector	117 (54%)	
	Other	55 (25%)	
Pregnancy			
	Full term pregnancy	188 (86%)	
	Preterm pregnancy	17 (8%)	
	Protracted pregnancy	13 (6%)	
Feeding			
	Breastfeeding	113 (52%)	
	Breast and bottle feeding	72 (33%)	
	Bottle feeding	31 (14%)	
	Other	2 (1%)	
Number of previous children			
	0	121 (56%)	
	1	73 (33%)	
	2	13 (6%)	
	3	1 (1%)	
	Not answered	10 (4%)	

*Note*: *n* = 246

### Measures

#### Work-family balance

Work-family balance [43, 44] is the instrument used to evaluate the perception of being able to successfully negotiate the role expectations and accomplish family and work responsibilities. This self-report questionnaire consists of 6 items (e.g. “My co-workers and members of my family would say that I am meeting their expectations”) on a 5-point Likert scale from 1 “strongly disagree” to 5 “strongly agree” that allows evaluating the perceived success in meeting expectations of other people (i.e., co-workers, supervisors, family members) at work and in the family [43]. Previous studies using this scale supported its psychometric properties [43, 44]. In this study, the level of reliability was adequate (α = 0.87) and a CFA indicated acceptable model fit, χ^2^ [9] = 13.40;, CFI = 0.99, NNFI = 0.99, RMSEA (90% CI) = 0.05 ( 0.00, 0.10), SRMR = 0.05.

#### Need Satisfaction

The Basic Psychological Need Satisfaction and Frustration Scale (BPNSFS; 33, 45) is the instrument used to assess need satisfaction. This self-report questionnaire consists of 24 items on a 5-point Likert scale from 1 “Not true at all” to 5 “Completely true”, that allow the evaluation of the satisfaction and frustration of the three basic psychological basic needs defined by self-determination theory of autonomy (i.e., “I feel my choices express who I really am”), relatedness (i.e., “I experience a warm feeling with the people I spend time with”), and competence (i.e., “I feel competent to achieve my goals”). In the present research contribution, an overall score of satisfaction of the three basic psychological needs was obtained, calculating as reverse all the items of frustration [35]. Previous studies using this scale supported its psychometric properties [33, 45]. In this study, the reliability coefficient was adequate (α = 0.92) and a CFA indicated acceptable model fit, χ^2^ (237) = 213.47; CFI = 1.00, NNFI = 1.00, RMSEA (90% CI) = 0.00 ( 0.00, 0.02), SRMR = 0.06.

#### Depression

The Center for Epidemiologic Studies Depression Scale (CES-D; [46]) is a self-report instrument that is designed to measure current symptoms of depression. In this study, the 5-item (e.g. “ I Felt depressed”) CES-D version [47] was used on a 4-point Likert scale from 1 “rarely or none of the time” to 4 “most or all of the time” that have shown good psychometric properties [47]. Higher scores indicated a higher frequency of depressive symptoms during the last weeks. The reliability coefficient was adequate (α = 0.80) and a CFA indicated acceptable model fit, χ^2^ (5) = 2.92; CFI = 1.00, NNFI = 1.00, RMSEA (90% CI) = 0.00 ( 0.00, 0.07), SRMR = 0.03.

#### Sleep–wake problems

Mini Sleep Questionnaire (MSQ; [48]) is a self-report instrument used to evaluate sleep difficulty and excessive daytime somnolence. The instrument consists of 10 items on a 7-point Likert scale from 1 “Never” to 7 “always”. This self-report scale included two dimensions: five items for the sleep difficulty (e.g. “Have you ever had trouble falling asleep”) and four for the daytime somnolence (e.g. “In the morning, after waking up, you felt tired). The Italian validation study have supported the psychometrics characteristics of the MSQ [48] and have shown that one item (i.e., snoring) did not load on any dimension and therefore it was excluded from the computation of the questionnaire's scores. The level of global reliability was adequate (α = 0.85) and a CFA indicated acceptable model fit, χ^2^ (26) = 42.31; CFI = 0.99, NNFI = 0.99, RMSEA (90% CI) = 0.05 ( 0.02, 0.08), SRMR = 0.07.

#### Vitality

The subject vitality scale (SVS; [49, 50]) is the instrument used to assess the state of subjective vitality. Specifically, it refers to a general experience of energy characterized by a sense of activation and vigor that is considered an integral part of the full human functioning and of the eudaimonic well-being [31, 49]. The version used in this study [50] consists of 6 items on a 7-point Likert scale from 1 “strongly disagree” to 7 “strongly agree”. (e.g. “I feel alive and vital”) that have shown good psychometric properties [50]. In this study, the level of reliability was adequate (α = 0.90) and a CFA indicated acceptable model fit, χ^2^ (9) = 2.68; CFI = 1.00, NNFI = 1.00, RMSEA (90% CI) = 0.00 ( 0.00, 0.00), SRMR = 0.02.

#### Anger

The Patient Reported Outcome Measurement Information System (PROMIS) Emotional Distress Scales—LEVEL 2—Anger—Adult (PROMIS Emotional Distress—Anger— Short Form; [51, 52]) is a self-reporting scale that evaluates angry mood. This scale is included in the DSM-5—Level 2—assessments of the Patient Reported Outcome Measurement Information System (PROMIS) banks items founded by the NIH [51]. This instrument consists of 5 items on a 5-point Likert scale from 1 “strongly disagree” to 5 “strongly agree”. (e.g. “I feel Angry”) and have shown good psychometric characteristics [51]. The reliability coefficient was adequate in this study (α = 0.86) and a CFA indicated acceptable model fit, χ^2^ (9) = 13.40;, CFI = 0.99, NNFI = 0.99, RMSEA (90% CI) = 0.05 ( 0.00, 0.10), SRMR = 0.05.

### Data analysis

The Statistical Package for Social Science (IBM SPSS Statistics 21) was used to conduct descriptive statistics (Mean, Standard Deviations, Skewness and Kurtosis), and Pearson correlations were used to investigate the possible association between the variables. The “lavaan” package of R with the implementation of R studio (version 4.0.4) was used to conduct CFA and SEM. The CFA for the instruments used in this study and reported in the method section were conducted with the unweighted least squares (ULS) in consideration of the ordinal nature of the items [53]. The hypothesized models with latent variables were tested using SEM with the Maximum Likelihood (ML) and a 5000 resample of bootstrapped estimates was used to test the direct and indirect associations. Mediation was tested by interpreting the 95% bootstrapped upper and lower confidence intervals (CIs) of indirect associations, and if the CIs of the indirect associations did not include zero, they were considered significant. Furthermore, the indicators of the latent variables for need satisfaction were represented by the scores of the three needs (autonomy, competence, and relatedness), while sleep–wake problems were represented by the two subscales of this measure (sleep difficulty, daytime somnolence). Depression and anger were represented by all their respective five items, while vitality and work-family balance were represented by three composite scores (parcels) each one created by the average of the respective scale item scores. In order to examine the relations between latent variables and their corresponding indicators, three different nested models were tested: 1) a one single factor model; 2) a three correlated factors model (predictors, mediators, outcomes); 3) the hypothesized measurement model, containing six separate correlated factors. The modes were compared on the basis of χ2 difference test and the differences in CFI. Overall, criteria for the goodness of the models were examined with the chi-square test (significant χ2 indicate a poorly fitting model), the comparative fit index (CFI ≥ 0.90), the non-normed fit index (NNFI ≥ 0.90), the standardized root mean residual (SRMR ≤ 0.08), and the root mean square error of approximation (RMSEA ≤ 0.08).

## Results

### Preliminary analysis

Descriptive statistics and correlation analyses for the study’s variables were reported in Table 2 and all the variables have shown levels of skewness and kurtosis within the range of ± 1, suggesting a normal distribution. Overall, the correlations were all statistically significant, with the work-family balance that was positively correlated with need satisfaction and vitality, while it was negatively correlated with sleep–wake problems, depression and anger. Furthermore, need satisfaction was positively correlated with vitality and negatively correlated with sleep–wake problems, depression and anger. Finally, sleep–wake problems, depression and anger were all positively correlated with each other.

**Table 2:** Descriptive statistics and correlations

	M	SD	Skewness	Kurtosis	α	1	2	3	4	5
1. Work-family balance	3.90	.72	-.69	.88	.87					
2. Need satisfaction	3.86	.63	-.49	-.28	.92	.52^**^				
3. Sleep–wake problems	31.92	10.14	.03	-.62	.85	-.25^**^	-.39^**^			
4. Depression	2.29	.76	.24	-.56	.80	-.28^**^	-.49^**^	.45^**^		
5. Vitality	4.43	1.26	-.26	-.24	.90	.48^**^	.53^**^	-.39^**^	-.40^**^	
6. Anger	2.18	.97	.66	-.22	.86	-.27^**^	-.56^**^	.29^**^	.45^**^	-.39**

^**^ p < .01, *p < *.05*

### Measurement model

Three different nested measurement models were tested in order to examine the relations between latent variables and their corresponding indicators. The model with only a single latent variable defined by all the indicators showed poor goodness of fit indexes, χ2(189) = 1140.54, *p* < 0.01, CFI = 0.58, NNFI = 0.54, RMESA (90% CI) = 0.15 (0.14, 0.16), SRMR = 0.11. Similarly, the intermediate model developed by three latent variables (a first factor by the three indicators of the work-family balance, the second factor by the three indicators of basic need satisfaction, and the third factor by the indicators of the sleep–wake problems, depression, vitality and anger), showed poor goodness of fit indexes, χ2(186) = 898.57, *p* < 0.01, CFI = 0.69, NNFI = 0.65, RMSEA (90% CI) = 0.13 (0.12, 0.14), SRMR = 0.10. Finally, the hypothesized measurement model containing six separate correlated factors (Table 3) showed good fits, χ2(174) = 306.26, *p* < 0.01, CFI = 0.94, NNFI = 0.93, RMSEA (90% CI) = 0.06 (0.05, 0.07), SRMR = 0.05, and there was a remarkable improvement compared to model 1, Δχ2(15) = 834.29, *p* < 0.01, ΔCFI = 0.40, and model 2, Δχ2(15) = 592.12, *p* < 0.01, ΔCFI = 0.25.

**Table 3:** Hypothesized measurement model

Latent Variable	Indicator	Standardized loadings	Standardized residuals
Need satisfaction	Subscale Autonomy	0.79	0.38
Need satisfaction	Subscale Competence	0.79	0.38
Need satisfaction	Subscale Relatedness	0.70	0.52
Work-family balance	Parcel Work-family balance 1	0.84	0.30
Work-family balance	Parcel Work-family balance 2	0.85	0.27
Work-family balance	Parcel Work-family balance 3	0.71	0.50
Sleep–wake problems	Subscale Daytime somnolence	0.84	0.29
Sleep–wake problems	Subscale Sleep difficulty	0.70	0.51
Depression	Item Depression 1	0.81	0.34
Depression	Item Depression 2	0.43	0.82
Depression	Item Depression 3	0.75	0.44
Depression	Item Depression 4	0.77	0.41
Depression	Item Depression 5	0.59	0.65
Vitality	Parcel Vitality 1	0.84	0.29
Vitality	Parcel Vitality 2	0.90	0.20
Vitality	Parcel Vitality 3	0.84	0.29
Anger	Item Anger 1	0.72	0.49
Anger	Item Anger 2	0.84	0.30
Anger	Item Anger 3	0.64	0.59
Anger	Item Anger 4	0.75	0.44
Anger	Item Anger 5	0.80	0.36

### Direct and indirect associations

**Fig. 2:**
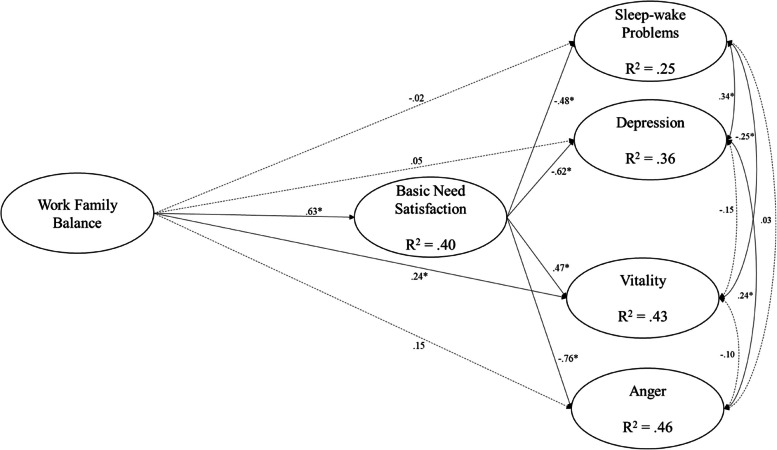
Structural equation model. Note: Coefficients shown are standardized path coefficients. Dotted line represents not significant associations. * *p* < .05

The hypothesized model with latent variables was tested using SEM with the Maximum Likelihood (ML), showing good fit indices, χ2(174) = 306.26, *p* < 0.01, CFI = 0.94, NNFI = 0.93, RMSEA (90% CI) = 0.06 ( 0.05, 0.07), SRMR = 0.05, and allowing the estimation of associations among the study variables (Fig. 2). Examination of the direct associations (Table 4) has shown positive associations from the work-family balance to the need satisfaction (*b* = 0.62, 95% CI [0.48, 0.76], *p* < 0.001, β = 0.63). Need satisfaction, in turn, was negatively associated with sleep–wake problems (*b* = -3.42, 95% CI [-5.36, -1.71], *p* < 0.001, β = -0.48), depression (*b* = -0.76, 95% CI [-1.08, -0.49], *p* < 0.001, β = -0.62), anger (*b* = -1.09, 95% CI [-1.51, -0.76], *p* < 0.001, β = -0.76), and positively associated with vitality (*b* = 0.83, 95% CI [0.43, 1.25], *p* < 0.001, β = 0.47). Additionally, the work-family balance was significantly associated with vitality (*b* = 0.42, 95% CI [0.07, 0.79], *p* = 0.023, β = 0.24). The other direct associations were not significant. Estimation of indirect associations (Table 4) through the implementation of the bootstrapping approach have shown significant results from work-family balance to the sleep–wake problems (*b* = -2.11, 95% CI [-3.37, -1.01], *p* < 0.001, β = -0.31), depression (*b* = -0.47, 95% CI [-0.70, -0.28], *p* < 0.001, β = -0.39), vitality (*b* = 0.51, 95% CI [0.25, 0.79], *p* < 0.001, β = 0.30), and anger (*b* = -0.67, 95% CI [-0.94, -0.44], *p* < 0.001, β = -0.48), through need satisfaction.

**Table 4:** Direct and Indirect Associations

	*b*	*p*	Lower CI 95%	Upper CI 95%	β
*Direct Associations*					
Work-Family Balance – > Need Satisfaction	0.62	< .001	0.48	0.76	0.63
Need Satisfaction – > Sleep–Wake Problems	-3.42	< .001	-5.36	-1.71	-0.48
Work-Family Balance – > Sleep–Wake Problems	-0.16	0.87	-1.93	1.68	-0.02
Need Satisfaction – > Depression	-0.76	< .001	-1.08	-0.49	-0.62
Work-Family Balance – > Depression	0.07	0.66	-0.22	0.35	0.05
Need Satisfaction – > Vitality	0.83	< .001	0.43	1.25	0.47
Work-Family Balance – > Vitality	0.42	0.02	0.07	0.79	0.24
Need Satisfaction – > Anger	-1.09	< .001	-1.51	-0.76	-0.76
Work-Family Balance – > Anger	0.21	0.17	-0.10	0.52	0.15
*Indirect association *via* Need Satisfaction*					
Work-Family Balance – > Sleep–Wake Problems	-2.11	< .001	-3.37	-1.01	-0.31
Work-Family Balance – > Depression	-0.47	< .001	-0.70	-0.28	-0.39
Work-Family Balance – > Vitality	0.51	< .001	0.25	0.79	.030
Work-Family Balance – > Anger	-0.67	< .001	-0.94	-0.44	-0.48

*Note*: Lower and Upper 95% Coefficient Intervals are unstandardized values

To provide a more conservative test of the primary hypothesis, the same model was tested controlling for continuous background variables (age of mothers, months of age, weekly hours of work, and numbers of children). The model with controlling variables showed good fit indices, χ2(234) = 385.07, p < 0.01, CFI = 0.94, NNFI = 0.92, RMSEA (90% CI) = 0.05 ( 0.04, 0.06), SRMR = 0.05, and all the previous associations remain significant after accounting for the background variables. Furthermore, examining the paths from the background variables to the study variables, mothers' working hours was positively associated with sleep–wake problems (*b* = -0.12, 95% CI [-0.03, 0.21], *p* = 0.007, β = 0.21), while all the other associations were not significant.

## Discussion

Results provide valuable insights into the dynamics between work-family balance and psychological outcomes through the lens of SDT and extend the relevance of psychological needs for autonomy, competence, and relatedness for working mothers with children aged between 1 to 36 months that it is a challenging period of adjusting to motherhood and balancing work responsibilities with childcare.

The positive association observed between work-family balance and need satisfaction is fully aligned with SDT's emphasis on the importance of supportive environments in facilitating need satisfaction [31], especially for mothers navigating the demands of both work and childcare. When mothers with infants and toddlers, experience a balance between their work and family responsibilities, they are more likely to feel competent in managing their roles (competence satisfaction), they could feel a greater sense of choice in their activities (autonomy satisfaction) and they may be more likely to feel appreciated both at home and at work (relatedness satisfaction).

Regarding the second aim of this study, findings revealed a negative relationship between the satisfaction of basic psychological needs and anger, depression and sleep–wake problems, and a positive association with vitality. These results are fully in line with previous studies, which have consistently highlighted similar associations between the satisfaction of basic psychological needs and emotional health of mothers during the perinatal phase [37, 38], and in working mothers [28, 29]. Furthermore, although several studies have shown a negative association between need satisfaction and sleep–wake problems [35, 36], this study provides an innovative contribution by examining this association in a sample of mothers with a child within the third year of age. This aspect is extremely important considering that mothers’ level of sleep satisfaction continues to be much lower than pre-pregnancy [11].

The estimation of the direct and indirect associations reveals the role of need satisfaction in the associations between work-family balance and various psychological outcomes. Specifically, the significant indirect effects suggest that work-family balance could be associated with sleep–wake problems, depression, vitality, and anger through the role of need satisfaction. Having the opportunity during the transition to motherhood to cultivate a harmonious balance between their professional and family roles could contribute to effectively managing both spheres allowing mothers to experience autonomy in organizing their lives, develop competence in juggling multiple responsibilities, and nurture meaningful relationships both at work and within the family, and in turn this could provide energy and vitality [31, 33]. At the same working mothers who struggle to meet the demands of their job while also attending to the needs of their child feels, constrained in their possibility to make decisions about their schedule, reducing their sense of autonomy, increasing the feelings of inadequacy of fulfilling both roles, undermining her sense of competence, create a sense of disconnection from colleagues and family members reducing the participation in activity and events. These experiences could create a sense of being overwhelmed, eroding emotional health, increasing anger [34], depression [33], fatigue and sleep–wake problems [35, 36] that circularly can further worsen the work-family balance by creating a vicious circle. Furthermore, the direct relationships between work-family balance and sleep–wake problems, depression, and anger become non-significant in the final model. While raw correlations initially showed significant relationships between work-family balance and these outcomes—aligning with previous research [23–25, 27]—these direct associations are no longer significant when accounting for indirect associations through basic psychological needs. This does not undermine the importance of work-family balance for the mental health of working mothers. Instead, it highlights that basic psychological needs play a crucial role in explaining how work-family balance impacts mental health. In addition to these indirect associations, the results of this study have also shown a direct association between work-family balance and vitality, confirming previous findings [23]. However, this result highlights that the association persists even after accounting for need satisfaction, suggesting that other mechanisms may still be unexplored. Overall, when working mothers feel they are effectively managing the demands of both work and family, it can directly boost their vitality. This may be because the harmonization of work and family contexts provides additional psychological, emotional, and social resources, which then positively influence their overall life experience.

## Study limitations

Overall, the findings of this study have shown the importance of addressing the unique challenges faced by mothers of young children in balancing their multiple roles using SDT as the theoretical framework. However, even if this study provides valuable insights regarding the associations between work-family balance, need satisfaction, depression, anger, vitality and sleep–wake problems in working mothers with a child within three years of age, several limitations should be acknowledged. Firstly, the cross-sectional nature of the study limits our ability to establish causal relationships among the variables. Future research employing longitudinal and/or experimental designs is warranted to provide a more comprehensive understanding of the underlying mechanisms. Secondly, reliance solely on self-report measures may introduce common method bias and response bias, potentially influencing the accuracy of our findings. In future research, the inclusion of objective measures, such as actigraphy to assess sleep–wake problems, can complement self-report measures, providing a more comprehensive and accurate understanding of participants' sleep patterns and mitigating potential biases associated with self-reports. Furthermore, although the results of the measurement models were generally adequate, some factor loadings were not particularly high (below 0.70). While there is no universally accepted criterion for judging the validity of an item as an indicator of a latent variable [54], it could be useful to conduct further investigation into the relationship between the indicators and the constructs examined in this study. Additionally, an important limitation of our study is the lack of exploration into dyadic mechanisms within couples, which may influence the dynamics of work-family balance and its effects. Future research should adopt a dyadic approach, examining the interplay between partners' perceptions and behaviours to provide a more nuanced understanding of how work-family dynamics impact parental well-being. Additionally, employing a dyadic approach can enhance the generalizability of findings by capturing the complexities of interpersonal relationships within diverse family contexts.

### Practical implications

Overall, the findings provide empirical support for the relevance of self-determination theory in understanding the mechanisms underlying the relationship between work-family balance and individual well-being, particularly among postpartum working mothers. Exploring the possible pathways through which work-family dynamics influence psychological outcomes via need satisfaction, the study contributes to our understanding of how organizations and policymakers can support new mothers' well-being during this critical life stage by fostering environments that facilitate autonomy, competence, and relatedness in both work and family domains.

Employers should consider and evaluate their employees' work-family balance levels on a regular basis. They should also effectively handle work context-related factors that have been shown to be important, such supervisor support and job control [26]. Indeed, employers can encourage informal behaviours that help, including supervisor assistance, and provide employees with more autonomy and control over their duties and work activities in order to enhance their feeling of balance. Additionally, organizations should encourage and implement new forms of flexibility in work organization, smart working, wellness programs, conciliatory vouchers, and childcare, increasing the use of parental leave, etc. [55].

Organizations should also make an investment in the promotion of work-family balance by putting in place suitable work-family policies [56, 57]. These policies can help employees manage relationships and tasks more effectively within the family setting, especially in light of the significance of family support [58, 59].

To this end, companies should support all aspects that encourage a sufficient balance, such as support in the workplace and in the home, and foster a work environment that is family-friendly in order to enable employees to efficiently manage the tasks within the various domains of life [60].

## Data Availability

The data supporting the conclusions of this study are available upon request to the first author, Sebastiano Costa.
